# Comparison of 3D and 2D area measurement of acute burn wounds with LiDAR technique and deep learning model

**DOI:** 10.3389/frai.2025.1510905

**Published:** 2025-02-27

**Authors:** Che Wei Chang, Hanwei Wang, Feipei Lai, Mesakh Christian, Shih Chen Huang, Han Yi Tsai

**Affiliations:** ^1^Graduate Institute of Biomedical Electronics and Bioinformatics, National Taiwan University, Taipei, Taiwan; ^2^Division of Plastic and Reconstructive Surgery, Department of Surgery, Far Eastern Memorial Hospital, New Taipei, Taiwan; ^3^Department of Electrical Engineering, National Taiwan University, Taipei, Taiwan; ^4^Department of Computer Science and Information Engineering, National Taiwan University, Taipei, Taiwan

**Keywords:** 2D segmentation, 3D segmentation, LiDAR, 3D measurement, anatomical location, curvature, deep learning—artificial intelligence

## Abstract

It is generally understood that wound areas appear smaller when calculated using 2D images, but the factors contributing to this discrepancy are not well-defined. With the rise of 3D photography, 3D segmentation, and 3D measurement, more accurate assessments have become possible. We developed an application called the Burn Evaluation Network (B.E.N.), which combines a deep learning model with LiDAR technology to perform both 2D and 3D measurements. In the first part of our study, we used burn wound templates to verify that the results of 3D segmentation closely matched the actual size of the burn wound and to examine the effect of limb curvature on the 3D/2D area ratio. Our findings revealed that smaller curvatures, indicative of flatter surfaces, were associated with lower 3D/2D area ratios, and larger curvatures corresponded to higher ratios. For instance, the back had the lowest average curvature (0.027 ± 0.004) and the smallest 3D/2D area ratio (1.005 ± 0.055). In the second part of our study, we applied our app to real patients, measuring burn areas in both 3D and 2D. Regions such as the head and neck (ratio: 1.641) and dorsal foot (ratio: 1.908) exhibited significantly higher 3D/2D area ratios. Additionally, images containing multiple burn wounds also showed a larger ratio (1.656) and greater variability in distribution. These findings suggest that 2D segmentation tends to significantly underestimate surface areas in highly curved regions or when measurements require summing multiple wound areas. We recommend using 3D measurements for wounds located on areas like the head, neck, and dorsal foot, as well as for cases involving multiple wounds or large areas, to improve measurement accuracy.

## 1 Introduction

Accurate burn area measurement is crucial for effective care management. The Lund and Browder chart (1942) (Taylor et al., 1943) and the Wallace rule of nines (1947) (Knaysi et al., 1968) became the most popular methods for estimating burn areas, with the Lund and Browder chart still widely used today. However, its accuracy depends on human judgment, and studies have shown significant discrepancies between estimates from referring units and burn centers (Harish et al., 2015; Baartmans et al., 2012). Interestingly, no evidence suggests that specialists provide more accurate estimates, as Parvizi et al. (2014) demonstrated that even experts show variability when evaluating the same burn cases.

In recent years, deep learning, a subset of machine learning, has gained prominence for its ability to extract data features through multiple convolutional layers, and it is widely used in both industrial and medical fields (Litjens et al., 2017; Ker et al., 2017). With proper training and well-labeled data, deep learning models can accurately segment burn wounds, converting segmented areas into %TBSA burned using pixel-to-pixel methods (Chang et al., 2021; Jiao et al., 2019; Chang et al., 2022), reducing the bias from human estimation. However, factors like photo conditions, wound distance, and obstructions (dressings, ointments, or hematomas) can affect segmentation accuracy, though these can be controlled. The primary challenge lies in the limitations of two-dimensional (2D) images, which generally underestimate wound areas compared to three-dimensional (3D) images. While some studies have used 3D imaging to estimate the area and volume of chronic ulcers, research on 3D imaging for burn wounds is limited, as burn wounds can take irregular shapes on convex surfaces, unlike chronic ulcers that are often elliptical or spherical on concave surfaces [an area formula 0.73 × L × W (L = length, W = width)] (Jørgensen et al., 2016).

Our initial goal was straightforward: given a 2D burn wound area (segmented from a 2D image) and the curvature of the extremities or trunk, what is the actual surface area in 3D? Since most practitioners rely solely on 2D images for burn wound documentation, our final aim is to develop a formula that accurately converts 2D wound areas to their corresponding 3D surface area measurements. We developed an application called the Burn Evaluation Network (B.E.N. in iOS), which combines a deep learning model with LiDAR technology to perform simultaneous 2D and 3D wound measurements. Through this app, we aim to explore the relationship between 2D and 3D measurements.

## 2 Development of applications

This study builds upon our previous work on burn wound segmentation and 3D coordination with LiDAR (Chang et al., 2021; Chang et al., 2022).

### 2.1 Data collection and model training

In accordance with regulations from Taiwan’s Ministry of Health and Welfare (MOHW), it is mandatory to document burn injuries using images or videos, which are stored electronically in hospital medical records and uploaded to the MOHW for insurance reimbursement. Verbal consent is obtained from patients or their families to use these records for wound documentation and research purposes. Our AI models were trained using a retrospective image collection from the Division of Plastic Surgery at Far Eastern Memorial Hospital. The study, approved by the hospital’s Ethics Committee (Approval Number 109037-F), began on May 1, 2020, and is ongoing with annual review. Data for model training was collected from January 2016 to December 2021.

To enhance diversity and segmentation performance, images were taken under different conditions, such as outpatient clinics, operation theaters or wards. These images were also taken by various devices, including cell phones and digital cameras. Images were excluded if wounds were covered by ointments or dressings, had undergone interventions like debridement or skin grafts, or were taken within 48 h of injury. Burn wounds were labeled using polygons in Labelme (Python 3.7), with two out of three burn surgeons co-labeling the images. One surgeon performed the initial labeling, and other surgeons reviewed the labels to identify necessary revisions. If significant disagreement arose, the image was discarded—typically due to factors like poor lighting or slight out-of-focus conditions.

A dataset of 10,088 labeled images was split into training, validation, and testing sets in a 7:2:1 ratio with 3-fold validation. Image augmentations like rotation, shifting, scaling, and contrast normalization were applied. Training was conducted on a server with eight NVIDIA TESLA V100 GPUs. Over a dozen model architectures and encoders were tired, including Mask RCNN, U-Net, PsPNet, and Inception. Ultimately, we selected DeepLabV3+ with ResNet101 as the encoder (He et al., 2016)—not because it delivered the absolute best performance, but because it offered the most balanced performance for practical, real-world use. The model was further converted to TensorFlow Lite for mobile deployment.

### 2.2 Combining LiDAR

LiDAR and digital cameras have been widely used in fields like archaeology, agriculture, and automotive industries (Dai et al., 2022; Debnath et al., 2023). LiDAR (Light Detection and Ranging) creates depth maps, and Apple has integrated this technology into devices like the iPhone 12 Pro and iPad Pro. We developed the Burn Evaluation Network (B.E.N.) app, available on the Apple Store, which uses our mentioned DeeplabV3+ with ResNet101 model for wound segmentation. Our app captures 2D images, segments them using the AI model, and integrates the 2D segmentation with the depth map to produce 3D segmentation results. With the 2D images from the camera and the depth data from LiDAR, we can convert the information into 3D real-world coordinates using Equation 1, where $m_{\mathrm{image}}$ is the 2D coordinate vector [*u v l*]^T^ of the image and *M*_world_ is the 3D coordinate vector [*x y z l*]^T^ of the real world wound, *K* is the camera intrinsic matrix, and [*R*∣*t*] is the camera extrinsic matrix.

**Figure 1:**
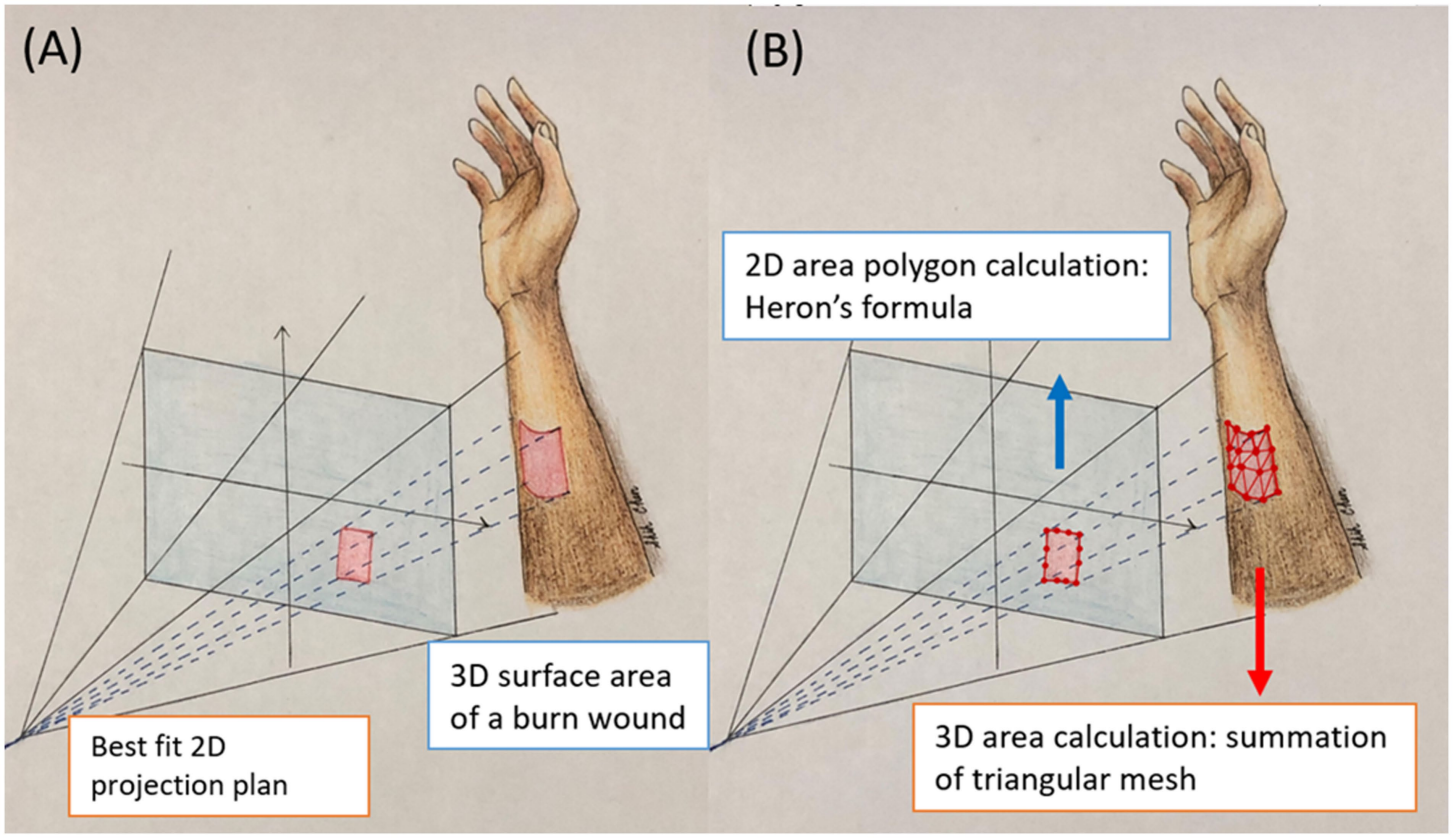
This illustration shows the 2D projection area and 3D surface area. **(A)** The projection area is flat 2D plan. The area is calculated with Heron’s formula. **(B)** The surface area is 3D curvature of the actual burn wounds. The area is calculated by summation of the area of small triangles.


(1) $$m_{\mathrm{image}}=K\left[R|t\right]M_{\mathrm{world}}\text{.}$$

The camera intrinsic matrix allows you to transform 3D camera coordinates to 2D image coordinates on an image plane using the pinhole camera model by Equation 2. The values *f_x_* and *f_y_* are the pixel focal lengths; *o_x_* and *o_y_* are offsets of the principal point from the top-left corner of the image frame. Since $m_{\mathrm{image}}$ is known and the *z* value of *M*_camera_ can be replaced by the depth information acquired by LiDAR, the remaining *x*, *y* values of *M*_camera_ can be resolved.


(2) $$m_{\mathrm{image}}=\;K\;M_{\mathrm{camera}},K=\left[\begin{array}{ccc} f_{x} & 0 & o_{x}\\ 0 & f_{y} & o_{y}\\ 0 & 0 & 1 \end{array}\right]\text{.}$$

The camera extrinsic matrix [*R*∣*t*] is a matrix relating to a camera’s position and orientation to a world or scene coordinate system, which is a matrix concatenation of a 3 × 3 rotation matrix *R* and 3 × 1 column vector translation *t*. Once we obtain $M_{\mathrm{camera}}$ we can use the camera extrinsic matrix to transfer from 3D camera coordinates into 3D real world coordinates by Equation 3


(3) $$M_{\mathrm{camera}}=\left[R|t\right]M_{\mathrm{world}}\text{.}$$

### 2.3 Calculation of 2D projection area

After the burn wound borders are segmented into polygons, the steps to calculate the projected area of the burn wounds are as follows:

- Obtain the corresponding 3D coordinates of the wound area through real-world coordinate conversion.
- Identify all contour points of the wound area in 3D space.
- Apply Martin Newell’s method to find the best-fit 2D plane.
- Project the contour points onto the 2D plane.
- Calculate the area of the wound contours on the best-fit 2D plane using Heron’s formula.

Although the projected area is in 2D, the best-fit plane is determined by the normal vector of the 3D surface. This approach addresses the challenge that users may not always hold the camera’s CCD parallel to the burn wounds, especially when the wound surface is complex or uneven.

Unlike traditional 2D methods that require the camera to be perpendicular to the wound bed, our approach only requires capturing the entire wound in a 3D view to derive the best-fit 2D plane.

### 2.4 Calculation of 3D surface area

It is important to note that the borders of the burn wound in 3D are identical to those in 2D, as they are segmented using the same model. However, while the 2D projection represents a flat surface, the 3D surface area accounts for the curvature of the actual burn wounds. To calculate the 3D surface area of a burn wound, we approximate the true curved surface using numerous small triangles (Figure 1). These triangles are formed by connecting three adjacent pixels within the wound area. The triangulation process involves scanning all 3D points of the wound from the top-left to the bottom-right. The total burn area is the integration of the areas of all small triangles. The steps are as follows:

- Define the burn wound in the 2D image using the segmentation model.
- Obtain the corresponding 3D coordinates of the wound area through real-world coordinate conversion.
- Divide the wound area into small adjacent triangles to closely fit the actual wound surface.
- Calculate the area of each triangle using Heron’s formula.
- Sum the areas of all triangles.

**Figure 2:**
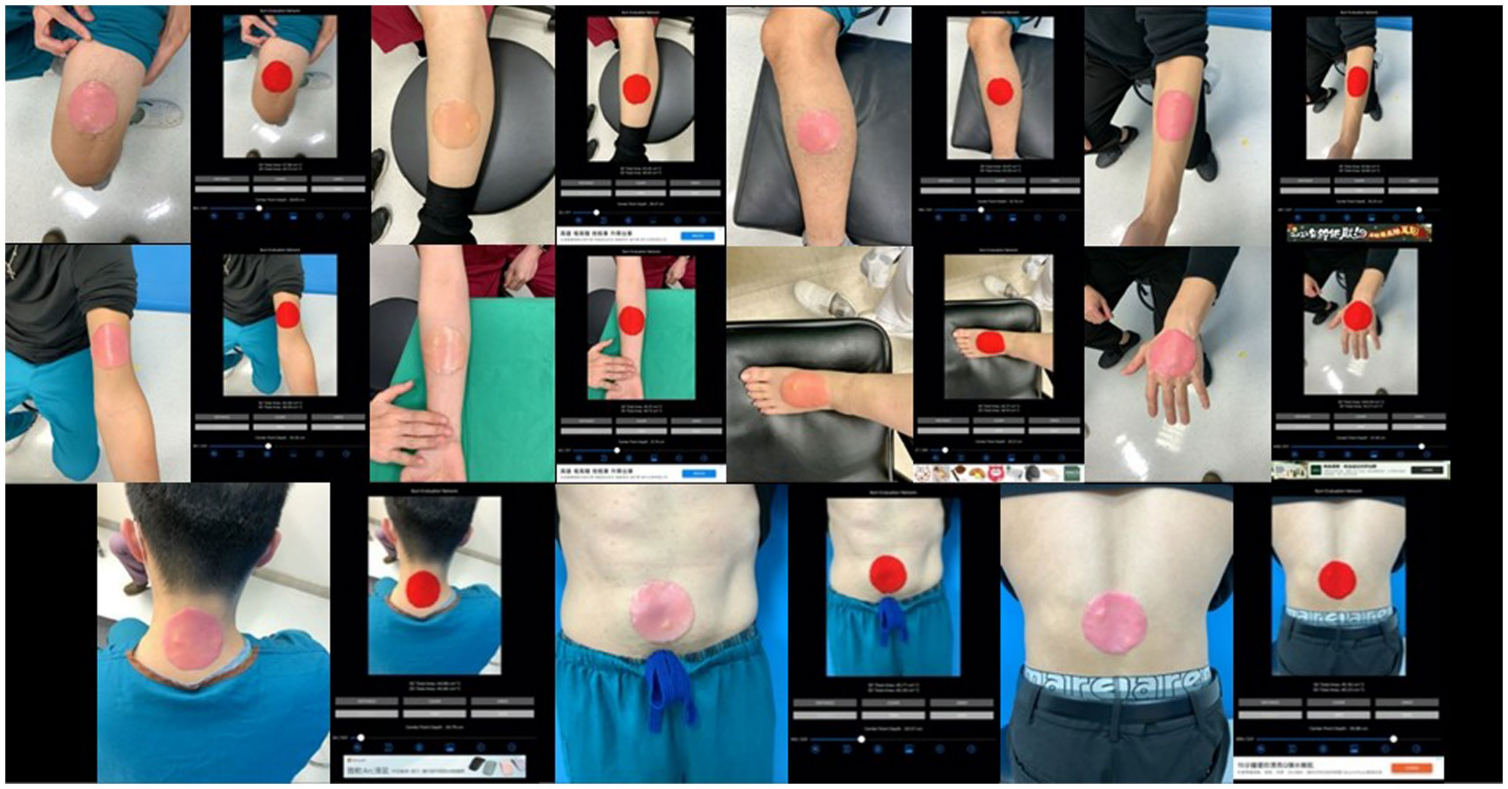
The segmentation of burn wound template on different anatomical locations.

When an image contains multiple burn wounds, our app sums the 2D and 3D results separately.

In summary, for 2D areas measurements, the polygon areas were calculated using Heron’s formula. For 3D surface areas measurements, the depth map was integrated with the polygon data to create surfaces. These surfaces were divided into small triangles by connecting adjacent pixels in order. The total 3D surface area was then calculated by summing the areas of all the small triangles.

## 3 Method and results

### 3.1 Part I: simulation study

The gold standard for 3D area measurement involves placing a transparent film over a burn wound and marking its edges. However, this method is less tolerable for patients with acute burns, as it requires direct application solely for measurement purposes. To address this limitation, we employed a burn wound template as a substitute to mimic real burns. Crafted by a professional prop maker, the template underwent testing with over 40 materials to accurately simulate burn wounds and challenge our AI models.

Initial trials with single-tone cardboard and cellophane, mimicking superficial burns, lacked the texture required for precise segmentation. Body paint crayons offered some improvement but lacked consistency. Ultimately, scar wax with varied color combinations proved most effective, enabling reliable segmentation by the app. Scar wax, composed of microcrystal wax, silicone, petroleum, and iron oxides, demonstrated excellent reusability due to its pliability and slight transparency.

The burn wound template must not be too large to require multiple images, avoiding issues with picture collaging. The template is round, sized at 1/400 of the average body surface area of an adult (17,000 cm^2^), approximately 43 cm^2^, allowing it to fit on any adult extremity and be captured in a single image. Round templates (43 cm^2^) were placed on the extremities and trunk of healthy participants. The app was then used to capture images, segment the templates, and measure both 2D projection and 3D surface areas (Figure 2).

**Figure 3:**
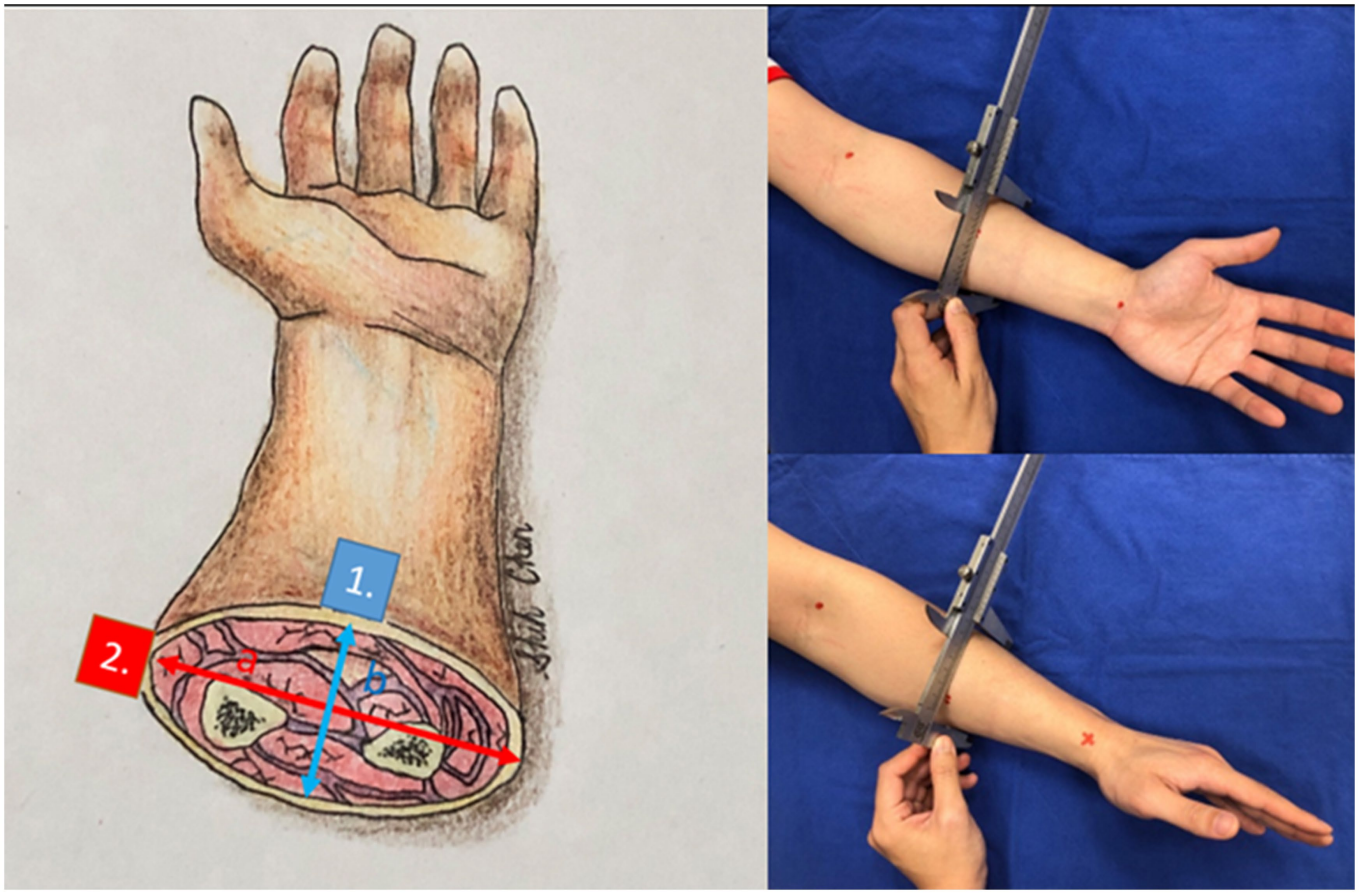
The cross-sections of the forearm and other anatomic locations have hypothesized an ellipse. Point 1 (blue) has a difference in curvature from point 2 (red). We gauged the long axis and short axis with a caliper.

After confirming that the 3D segmentation results closely match the ground truth (43 cm^2^) across all anatomical locations, we established that 3D segmentation can serve as a reliable surrogate for real wounds. Our next objective was to determine whether curvature affects the 3D-to-2D ratio for a given wound size using the same wound template. We gauged the diameters of the sites where the templates were placed, including various locations on the forearm, arm, leg, thigh, trunk, and neck. We hypothesized the cross-sections of these areas as ellipses and measured their long and short axes with a caliper and tape measure. In Figure 3 the curvature of the volar side of the forearm (point 1) is *b*/*a*^2^ whereas the curvature of the volar side of the forearm (point 2) is *a*/*b*^2^. The volar side of the forearm is flatter and has smaller curvature. If the cross-section of the extremity is almost a circle, which means (*a* ≈ *b* = *r*), the curvature is 1/*r*.

**Figure 4:**
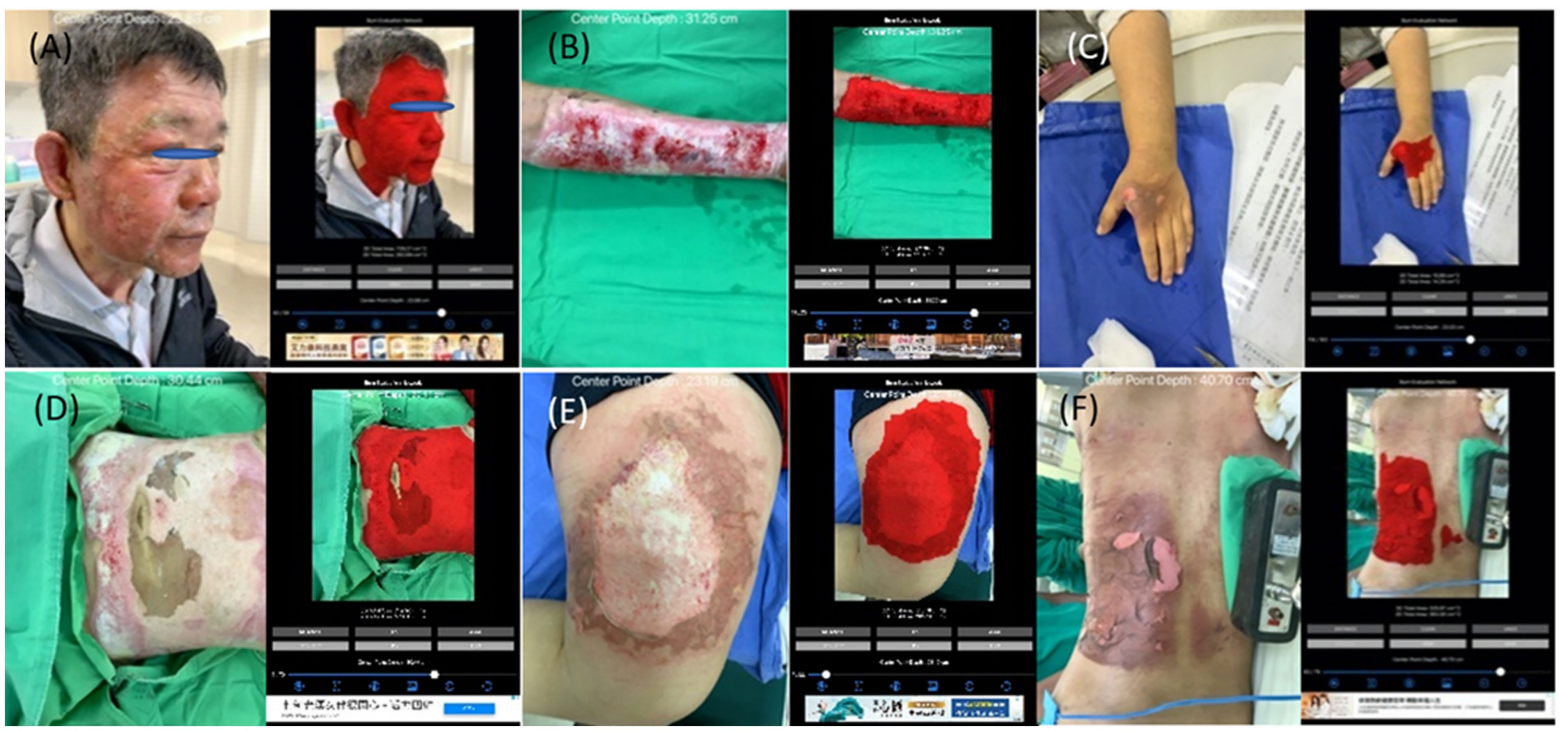
The collages of images of burn wounds on a single anatomical site with the segmentation result by B.E.N. **(A)** Head & neck; 3D area 729.27 cm^2^, 2D area 262.68 cm^2^. **(B)** Forearm & arm; 3D area 259.98 cm^2^, 2D area 133.57 cm^2^. **(C)** Hand; 3D area 16.10 cm^2^, 2D area 14.42 cm^2^. **(D)** Anterior trunk; 3D area 745.46 cm^2^, 2D area 504.27 cm^2^. **(E)** Thigh; 3D area 302.61 cm^2^, 2D area 274.38 cm^2^. **(F)** Posterior trunk; 3D area 525.97 cm^2^, 2D area 363.3 cm^2^.

### 3.2 Results of simulation study

The simulation study has received approval from the Research Ethics Review Committee of Far Eastern Memorial Hospital (Number 111084-E). This study included 10 female colleagues with normal BMI (18.3 to 22.4) and 11 male colleagues, including authors, with BMI (22.1 to 24.5). All participants provided written consent to measure the cross-sections of extremities, trunk, and neck, and to test burn templates. We measured the long and short axes of these anatomical locations and calculated their curvatures. A circular burn wound template (43 cm^2^) was placed on the measured locations, and images of the simulated wounds were captured 25–30 cm above the site using our app. The app also segmented the wounds, providing 3D surface area and 2D projection area measurements.

Our final model (DeeplabV3+ with ResNet101) demonstrated robust performance across multiple test datasets, all of which consisted of images of real patients labeled by experienced plastic surgeons. The average performance metrics on the test datasets were as follows: precision 0.9076, recall 0.9006, accuracy 0.9846, *F*_1_ score 0.8938, IoU 0.8288, and Dice loss 0.1063. These results were consistent across repeated evaluations.

Table 1 presents the ratio of the 3D result to 43 cm^2^, the ratio of the 3D area to the 2D area, and the curvature of the body’s cross-section. For example, one author’s forearm had a cross-section with a long axis of 7.4 cm and a short axis of 7.0 cm. The calculated curvatures were 0.127 for the volar forearm and 0.151 for the radial forearm. The segmentation results for the volar forearm were 43.68 cm^2^ (3D) and 39.42 cm^2^ (2D), while for the radial forearm, they were 43.59 cm^2^ (3D) and 34.64 cm^2^ (2D).

**Table 1:** The final result of the simulation study (mean ± SD).

	Female group (*n* = 10)			Male group (*n* = 11)		
Location	3D area/GT^a^	3D area^b^/2D area^c^	Curvature	3D area/GT	3D area/2D area	Curvature
Forearm (volar)	1.075 ± 0.012	1.017 ± 0.306	0.138 ± 0.017	1.027 ± 0.052	1.055 ± 0.103	0.121 ± 0.020
Forearm (radial)	1.084 ± 0.023	1.220 ± 0.040	0.183 ± 0.021	1.077 ± 0.040	1.223 ± 0.345	0.154 ± 0.019
Arm (anterior)	1.018 ± 0.049	1.164 ± 0.078	0.154 ± 0.018	1.004 ± 0.076	1.119 ± 0.050	0.156 ± 0.015
Hand (dorsal)	0.982 ± 0.038	1.340 ± 0.087	0.037 ± 0.004	0.988 ± 0.060	1.402 ± 0.033	0.032 ± 0.004
Leg (anterior)	1.042 ± 0.014	1.113 ± 0.022	0.139 ± 0.028	1.031 ± 0.048	1.116 ± 0.035	0.105 ± 0.011
Leg (lateral)	0.999 ± 0.054	1.084 ± 0.039	0.077 ± 0.010	1.030 ± 0.052	1.064 ± 0.036	0.069 ± 0.007
Thigh (anterior)	1.077 ± 0.011	1.083 ± 0.010	0.099 ± 0.010	1.040 ± 0.046	1.100 ± 0.058	0.078 ± 0.009
Foot (dorsal)	1.078 ± 0.043	1.203 ± 0.035	0.104 ± 0.020	1.017 ± 0.055	1.135 ± 0.070	0.081 ± 0.009
Neck (nape)	1.011 ± 0.063	1.100 ± 0.053	0.114 ± 0.017	1.015 ± 0.046	1.108 ± 0.067	0.088 ± 0.006
Abdomen	1.056 ± 0.027	1.055 ± 0.031	0.027 ± 0.004	1.023 ± 0.051	1.062 ± 0.061	0.023 ± 0.003
Back (lower)	1.008 ± 0.046	1.005 ± 0.055	0.027 ± 0.004	0.992 ± 0.051	1.005 ± 0.050	0.023 ± 0.003

a Ground truth, the size of the burn wound template is 43 cm^2^.

b Segmentation result of 3D surface area of the burn wound template.

c Segmentation result of 2D projection area of the burn wound template.

For all locations, the ratios of the 3D surface area to 43 cm^2^ (ground truth) are very close to 1.0. In male participants, these ratios range from 0.988 to 1.077, while in female participants, they range from 0.982 to 1.084. This suggests that the 3D segmentation results can accurately represent the true size of a burn wound.

When examining the ratios of 3D to 2D area and the curvature of the limb and trunk, it is evident that curvature significantly influences the ratio. The results in Table 1 support our assumption that flatter surfaces yield ratios of 3D area/2D area closer to 1. Flatter surfaces correspond to smaller curvatures, with the back and abdomen exhibiting the flattest surfaces in both females and males. The smallest ratio is observed on the back (female: 1.005, male: 1.005). Conversely, locations with greater curvature, such as the radial forearm, show higher 3D/2D ratios, indicating that 2D segmentation tends to underestimate the surface area more significantly in highly curved regions.

The order of curvature across different anatomical locations is consistent between females and males, and the ranking of 3D/2D ratios from smallest to largest is nearly identical for both genders. Additionally, male participants generally have smaller curvatures than females at the same locations, likely due to the larger diameters of their extremities and trunk, resulting in flatter surfaces.

### 3.3 Part II: study of real patients

Since its development, our app has been routinely integrated into our medical workflow for photo documentation and burn wound measurements. The app captures both 2D and 3D images of burn wounds, automatically segmenting and measuring the wound areas using an AI model. The absolute area (in square centimeters) can be converted to TBSA% by also segmenting the patient’s palm, which represents 0.5% of the TBSA. However, this functionality is not included in the current study.

Then, we categorize image sets into two groups: wounds affecting a single anatomical site and those involving multiple anatomical locations. Based on our experience, wounds spanning multiple sites tend to show a greater discrepancy between 2D and 3D segmentation results, highlighting the need for 3D measurement to ensure more accurate assessments in these cases.

### 3.4 Results of study of real patients

A total of 1,426 sets of images of acute burn wounds were collected since December 2021 to December 2022. Every set of images comprised Y.bin, CbCr.bin, depth.bin, and 2D and 3D segmentation results as .jpg.

#### 3.4.1 Single location of burn wound

Among the group of a single site, we further classified image sets based on the anatomical locations of burn wounds as the simulation study. The number of images of different locations are listed as head & neck: 74 images, anterior trunk: 138 images, forearm & arm: 222 images, hand: 68 images, thigh: 208 images, leg: 202 images, foot: 113 images, posterior trunk: 56 images. In Figure 4, the collages presented images of burn wounds involving on single anatomic location. The segmentation results of the 3D surface area and 2D projection area by B.E.N. are adjacent to the original images.

**Figure 5:**
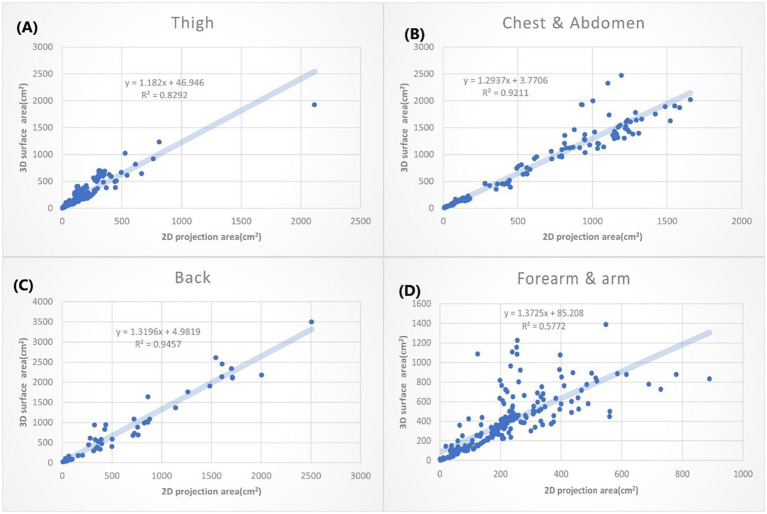
The *XY* plot of the 2D projection area and 3D surface area. These four locations show the small 3D/2D ratio (the slope of the regression). **(A)** Thigh: 1.182. **(B)** Chest & abdomen: 1.2937. **(C)** Back: 1.3196. **(D)** Forearm & arm: 1.3726.

**Figure 6:**
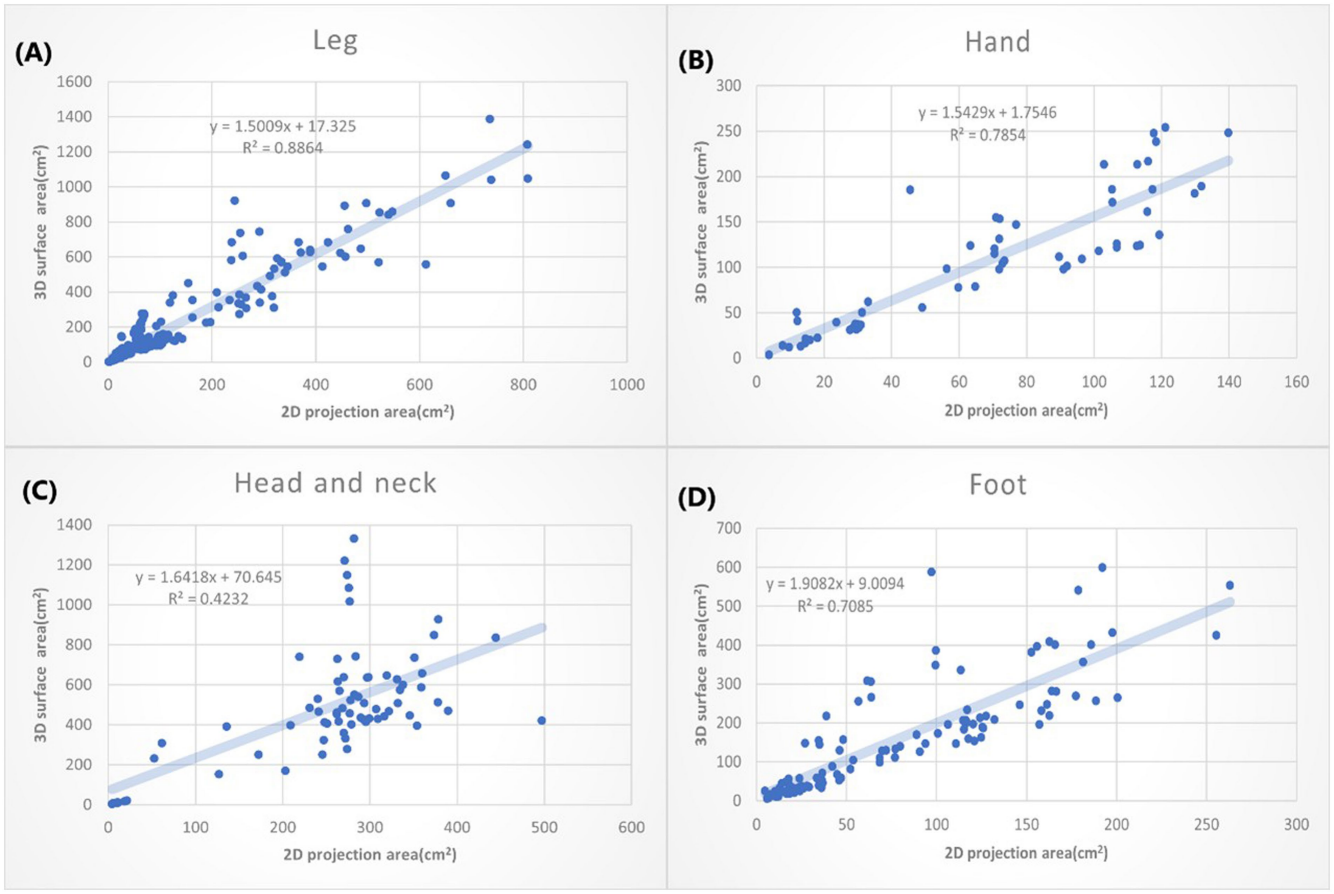
The *XY* plot of the 2D projection area and 3D surface area. These four locations show large 3D/2D ration. **(A)** Leg: 1.5009. **(B)** Hand: 1.5429. **(C)** Head & neck: 1.6418. **(D)** Foot: 1.9082.

According to different anatomical locations, we plotted the 2D projection area as *X* coordinate and the 3D surface area as *Y* coordinate. The linear regressions were calculated. The slope of the linear equation presents how many times the 3D surface area is to the 2D projection area average. The *R* squared (*R*^2^) suggests how good the relation of the 3D and 2D area could be explained by this linear equation. We listed the anatomic location by the order with small to large slopes in Figures 5, 6.

**Figure 7:**
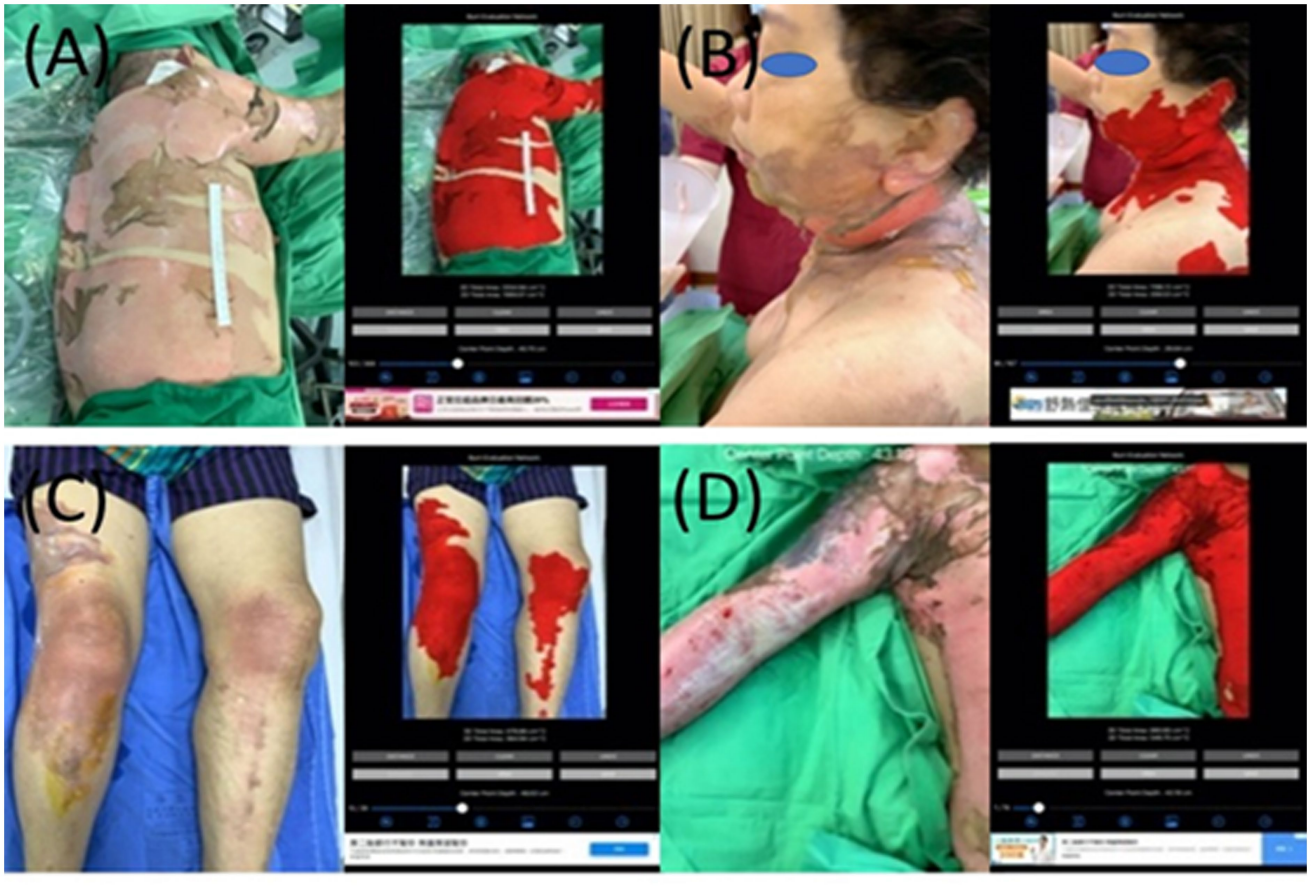
The collages of images of burn wounds in multiple locations with the segmentation results by B.E.N. **(A)** Back, lateral chest wall, and right upper extremity, 3D area 3,660.17 cm^2^, 2D area 1,113.65 cm^2^. **(B)** Cheek, ear, neck, back, and chest wall, 3D area 1,149.81 cm^2^, 2D area 204.24 cm^2^. **(C)** Bilateral knees legs and right thigh, 3D area 473.26 cm^2^, 2D area 350.75 cm^2^. **(D)** Right forearm and arm shoulder and chest wall, 3D area 890.90 cm^2^, 2D area 546.70 cm^2^.

The burn wounds on the thighs show the smallest slope (y=1.182x + 44.946, $R^{2}=0.8292$). That suggests the burn wounds on the thigh only had a slight difference when they are measured either by the 2D or 3D method. The second smallest slope is bun wounds on the chest wall and abdomen, followed by the back (Figure 5). That can be partially explained by that thigh and trunk of humans are relatively flat surfaces over the whole body. The burn wounds on the forearm and arm also slow the small slope (1.3726). However, the *R*^2^ is only 0.578, which means the results are still variable. It may depend on the locations of burn wounds on the forearm and arm. For example, the volar side of the forearm is quite flat, whereas the radial side has much curvature.

In Figure 6, the greatest slope is the burn wound on the foot (1.9082), followed by the head and neck (1.6418). That may suggest when we use the 2D methods to measure the burn wounds on the foot, we might underestimate the real size of the burn wound by nearly 50%. The great slope could be partially explained by the complex structure of the foot, head and neck. Moreover, the head and neck compose of so many unique structures, including the chin, submental, nose, and ears, which may not simplify as concave or convex. That further explained why the *R*^2^ of 3D area/2D area burn area on the head and neck is low (*R*: 0.4232).

#### 3.4.2 Multiple locations of burn wounds

There are 345 sets of images collected in the group of multiple locations. In Figure 7, all images capture burn wounds involving multiple anatomic locations, and the segmentation results of 2D and 3D by B.E.N.

**Figure 8:**
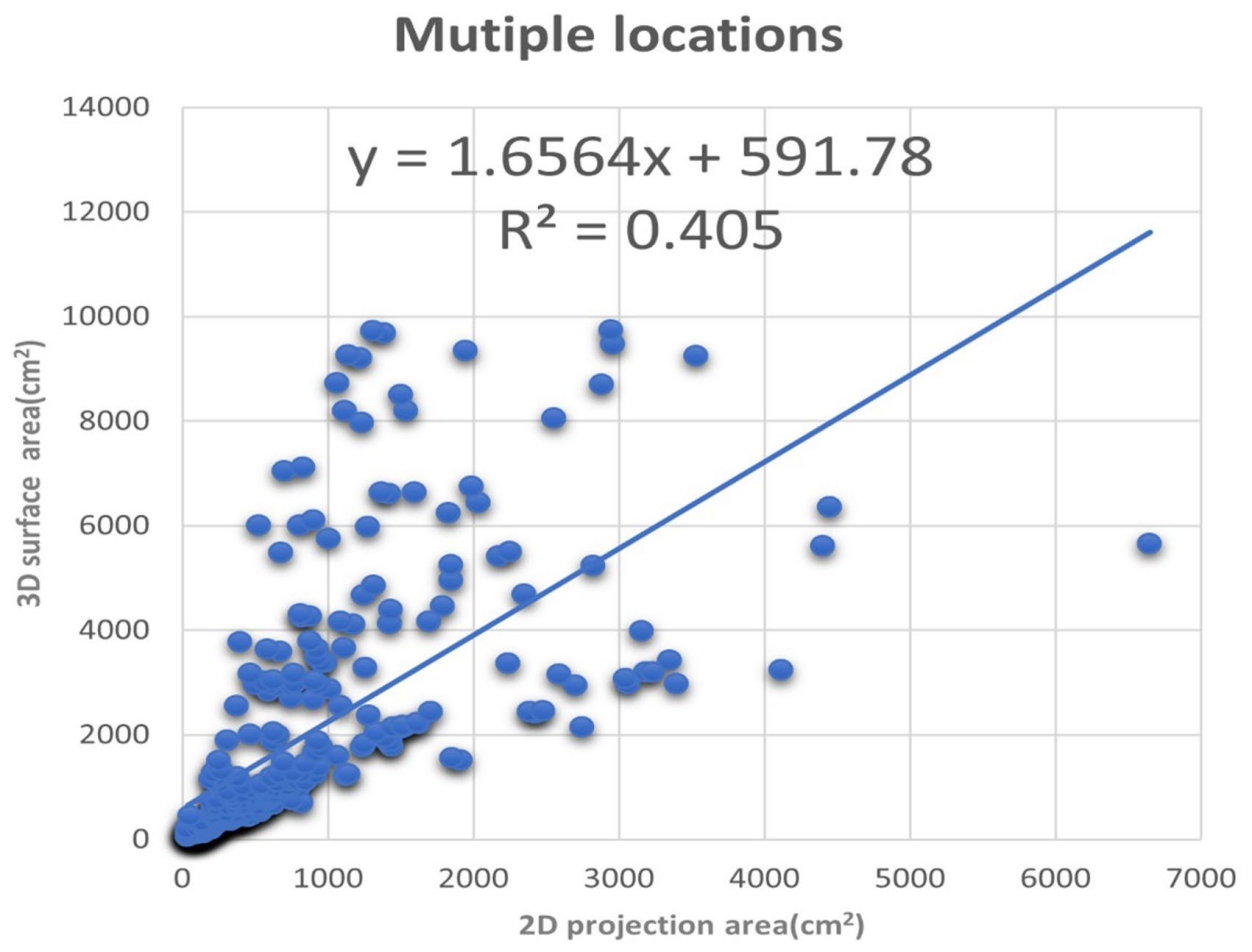
The *XY* plot of the 2D projection area and 3D surface area. These images of the burn wounds involve multiple anatomic locations.

We also plotted the 3D surface areas and 2D projection areas as *Y* and *X* coordinates in Figure 8. The regression of the plot is (*y* = 1.6564*x* + 591.78, *R*^2^ = 0.405). The slope is 1.6564, which means the average 3D surface area is 1.65 times than 2D projection area. The results suggest that if the burn area is estimated via 2D images of the major burn patient, such as using ImageJ, the true area will be seriously underestimated. For example, when we took a picture of a major burn patient in Figure 7, the actual 3D surface area is 3 times more than the projection area on a 2D image.

**Figure 9:**
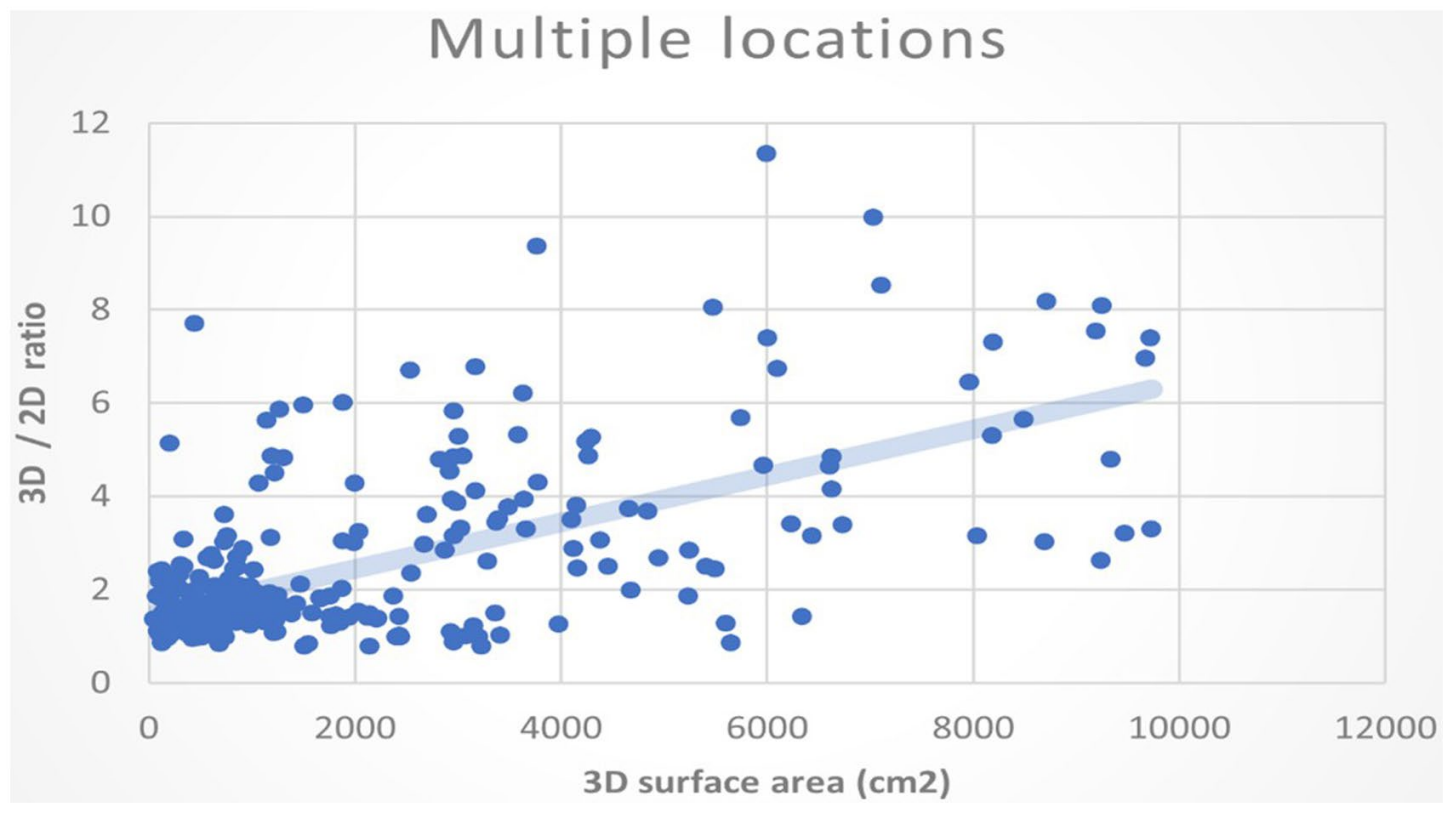
The relationship of the burn wound size to the ratio of 3D/2D area.

In addition, we would like to know whether the larger the burn area, the ratio of the 3D surface to the 2D area also increases. We assumed the deviation between 3D and 2D methods will augment when the overall burn area is large. We plotted the 3D surface area of the burn wound and the ratios of 3D to the 2D area as *XY* coordinate in Figure 9. The regression of the plot shows a positive slope. It indicates that as the actual area is large, the ratios of 3D/2D also increase. Using areas of 2D projection may highly underestimate the real size of burn wound.

## 4 Discussion

### 4.1 Mini review: from 3D photography to 3D segmentation to 3D auto measurement

Three-dimensional (3D) measurement has become an important topic in wound management. Before the rise of 3D photography, 2D measurement methods like the ruler method, manual or digital planimetry, and computerized stereophotogrammetry were mainstream (Langemo et al., 1998). These methods were widely studied for ulceration, with the ruler method showing deviations of up to 40% from the true size for oval wounds (Rogers et al., 2010; Langemo et al., 2008). However, the deviation of 2D methods in burn wounds has been less explored, as burn areas are often expressed as %TBSA and typically deviate from simple oval shapes.

### 4.2 3D photography

The advent of 3D measurement largely stems from advancements in 3D photography. Unlike traditional 2D images, 3D images capture RGB vectors in not just *XY*, but also a *Z*-coordinate system. Three common methods exist to acquire the *Z*-coordinate (depth map):

1. Stereo vision uses two or more cameras with known disparity to calculate the relative depth of objects through a “disparity map.” Stereo vision is a passive technology and does not require artificial lighting (O’Riordan et al., 2018).
2. Structured light projects laser light onto an object and measures the reflected light to determine shape and distance. This method can apply different wavelengths and phase shifts for greater detail and is an active technology (Geng, 2011).
3. Time of flight (ToF) uses lasers and sensors to calculate the distance of objects from the camera, forming a depth map. The use of LiDAR is common in this method (Behroozpour et al., 2017).

### 4.3 3D segmentation

3D photography sets the foundation for achieving accurate 3D measurements. Studies have demonstrated that 3D methods are more accurate than 2D techniques. Shah et al. (2013) found that 3D scanners using structured light had an 11% deviation in volumetric measurements, compared to 75% with the ruler method, 41% with acetate grid tracing, and 52% with 2D planimetric methods. Bowling et al. (2011) demonstrated that 3D measurement systems with manual labeling had acceptable intra-rater (3.3%) and inter-rater (11.9%) variations.

Commercial devices, such as the MAVIS system, also showed reduced standard deviation compared to transparency tracings (Plassmann and Jones, 1998). While these systems proved the efficacy of 3D measurement, they required special equipment, limiting daily accessibility. To address this, we developed an app for automatic 3D measurement of pressure ulcers on popular mobile devices like the iPhone and iPad (Liu et al., 2023). Our app demonstrated comparable results to traditional planimetry, which is less suitable for burn wounds, prompting us to design a simulation study to further validate the app’s accuracy.

### 4.4 3D measurement

3D measurement for acute burn wounds has received less attention compared to chronic ulcers. Before the integration of 3D photography, digital planimetry was considered the gold standard for measuring burn wound surface areas. However, digital planimetry requires the placement of a transparent grid over the wound, limiting its use to small areas (Kee et al., 2015). Stockton et al. (2015) showed that 3D photography was as accurate as digital planimetry in measuring burn wounds, with the added benefit of not requiring contact with the wound. While manual labeling of wound borders still introduces variability, Rashaan et al. (2018) demonstrated that 3D photography maintains good inter-rater reliability between different observers.

With the increasing availability of 3D scanners, commercial systems such as Intel RealSense have been applied to burn wound measurements. Bairagi et al. (2022) reported strong agreement between results from RealSense and other 3D wound measurement devices like LifeViz.

#### 4.4.1 Steps toward 3D measurement

To achieve 3D measurement, the process typically involves several steps:

1. 3D photography: Devices like those using stereophotogrammetry or structured light capture both the RGB image and a depth map for each pixel.
2. Conversion to 2D plan: Specialized software then converts the 3D surface data into a 2D plan, similar to a Mercator projection.
3. Manual labeling: Burn specialists label the borders of the wound on the 2D plan.
4. 3D measurement: Finally, the labeled areas are used to calculate the wound area with reference data.

Popular software tools for this process include Panasonic FZ-M1 with WoundCare Lite (Farrar et al., 2017), LifeViz with DermaPix, RealSense with Wound Measure, and Artec MHT with Artec 3D Studio.

Moreover, there are alternative methods that do not rely on 3D photography yet still achieve accurate results. BurnCase 3D, for instance, calculates 3D area measurements by mapping burn areas onto a corresponding 3D body model (Haller et al., 2009; Dirnberger et al., 2004). These body models are constructed using data collected from a diverse population, encompassing various ages, genders, weights, and heights. This approach significantly reduces human error compared to relying solely on the Lund and Browder chart.

### 4.5 Our progress:3D auto measurement

Building on previous work, we developed an app for iPhones and iPads equipped with LiDAR sensors, eliminating the need for additional 3D scanners or annotation software specifically for wound measurement. Our app streamlines the process of capturing 2D/3D photographs, performing 2D/3D segmentation, and automatically measuring both 2D and 3D areas.

Trained on over 10,000 well-labeled images of acute burn wounds, the app achieves high accuracy in wound segmentation. It ensures consistent results for wound borders, reducing the inter-rater and intra-rater variability typically associated with manual labeling. The app provides segmentation results for both 3D surface area and 2D projection area, enabling direct comparison between the two. This also allows us to study the influence of limb and trunk curvature on the 3D/2D area ratio.

DeeplabV3+ with ResNet101 demonstrated strong performance in both 2D and 3D segmentation tasks, achieving average scores of precision (0.9076), recall (0.9006), accuracy (0.9846), *F*_1_ score (0.8938), IoU (0.8288), and Dice loss (0.1063) on the test dataset. These results remained consistent across repeated evaluations, although performance slightly varied depending on the model configurations, encoders, and characteristics of the test datasets.

For straightforward cases, such as single burn wounds on extremities or the abdomen (e.g., scald burns), the model achieved precision and recall scores ranging from 0.95 to 0.98, surpassing human assessment as reported in our previous study (Chang et al., 2021). Conversely, in more complex scenarios involving multiple burn wounds within a single image, such as in patients with major burns, precision and recall scores decreased to approximately 0.85 to 0.89. These “challenging” images also posed significant difficulties for plastic surgeons during manual labeling, which is inter-rater variability.

### 4.6 Limitations

There are several limitations related to the hardware, software, and study. According to Apple Inc.’s specifications, the LiDAR sensor on iPhones and iPads functions optimally within a five-meter range. Its resolution of 192 × 256 may be insufficient, potentially leading to mapping inaccuracies during the 2D-to-3D transformation. For very small wounds, wounds on flat surfaces, or images taken from a distance, the 3D segmentation results may be indistinguishable from 2D results. However, these distance and resolution limitations are expected to improve with future device generations.

Our deep learning model was trained on over 10,000 images captured under various hospital lighting conditions. Segmentation accuracy decreases in poor lighting or non-hospital environments, and performance is also less reliable for patients with lighter skin tones. The simulation experiment was conducted on 21 colleagues with normal BMI, and the real-world study included 1,426 images of acute burn wounds. However, further data is needed to determine whether limb and trunk curvature affect measurements similarly in obese or underweight patients.

To address these issues, we continually update the model using incremental learning to enhance its adaptability. Our ultimate goal is to develop a formula that defines the relationship between curvature, 3D burn wound size, and 2D burn wound size.

## 5 Conclusion

To the best of our knowledge, this is the first study to compare 3D and 2D area measurements of acute burn wounds using the same device. We developed a deep learning-based application to automate burn wound segmentation, reducing the errors associated with manual labeling. Our app is designed for popular devices equipped with LiDAR sensors, such as the iPhone Pro and iPad Pro, allowing simultaneous capture of both 2D and 3D images. The app has been validated through both simulation studies and real patient applications for automated measurement.

Our study consists of two parts, following the approach of the pioneering study (Prieto et al., 2011). The first part employs “fake burns” in a simulation study to validate that our 3D segmentation results closely align with the gold standard, the “burn wound template.” This template serves as a substitute for the less tolerable method of applying transparent film directly to burn wounds. We demonstrated that flatter anatomical locations tend to exhibit smaller ratios of 3D to 2D wound areas. Although this observation is intuitive, our findings provide evidence to support it. For example, the back, with the smallest curvature (0.027 ± 0.004), showed the closest match between 2D and 3D measurements, with a ratio of 1.005 ± 0.055.

In the second part, we used the app to capture 2D and 3D images of real patients for medical records, along with providing 2D and 3D area measurements. The results showed that the smallest average 3D to 2D ratio was found in burn wounds on the anterior thigh, followed by the back and anterior trunk, where curvature is minimal. In contrast, wounds on the head and neck (ratio = 1.64), dorsal foot (ratio = 1.91), and those involving multiple locations (ratio = 1.65) exhibited larger ratios. These findings suggest that relying solely on 2D measurements may significantly underestimate the true size of burn wounds in these areas.

## Data Availability

The datasets presented in this article are not readily available because requests need to be submitted to our Research Ethics Review Committee. Requests to access the datasets should be directed to irb@mail.femh.org.tw.
